# Pattern and Appropriateness of Medicines Prescribed to Outpatients at a University Hospital in Northwestern Ethiopia

**DOI:** 10.1155/2017/3729401

**Published:** 2017-12-18

**Authors:** Fitsum Sebsibe Teni, Sewunet Admasu Belachew, Begashaw Melaku Gebresillassie, Eshetie Melese Birru, Befikadu Legesse Wubishet, Bethelhem Hailu Tekleyes, Bilal Tessema Yimer, Yonas Getaye Tefera

**Affiliations:** ^1^Department of Pharmaceutics and Social Pharmacy, School of Pharmacy, College of Health Sciences, Addis Ababa University, Addis Ababa, Ethiopia; ^2^Department of Clinical Pharmacy, School of Pharmacy, College of Medicine and Health Sciences, University of Gondar, Gondar, Ethiopia; ^3^Department of Pharmacology, School of Pharmacy, College of Medicine and Health Sciences, University of Gondar, Gondar, Ethiopia; ^4^Research Center for Generational Health and Ageing, Faculty of Health and Medicine, University of Newcastle, Callaghan, NSW, Australia; ^5^Department of Pharmaceutics, School of Pharmacy, College of Medicine and Health Sciences, University of Gondar, Gondar, Ethiopia

## Abstract

The study assessed the pattern and appropriateness of medicines prescribed to outpatients at Gondar University Referral Hospital in northwestern Ethiopia. An institution-based cross-sectional study was employed, through interviews and prescription reviews, among 346 patients at the outpatient pharmacy, from 2nd to 20th of May 2016. Data on sociodemographic profile of patients and medicines prescribed to them were collected. A mean of 1.72 medicines per encounter was prescribed, over a third of the total being anti-infectives. Patients were able to get about 85% of these medicines. An unskilled government employee would be required to work more than one and a half day to be able to afford the average priced medicine. Among prescriptions with two or more medicines, more than a third had at least one potential drug-drug interaction (PDDI), the commonest pair containing amoxicillin and doxycycline. Being male, being older (50–59 years), and increased number of medicines were associated with higher likelihood of PDDIs. In conclusion, the number of medicines prescribed per encounter was up to accepted standard. However, their availability fell short, together with considerable cost. Regarding appropriateness, a significant proportion of potential drug-drug interactions is identified and associated with patient's sex, age, and number of medicines prescribed.

## 1. Introduction

Globally, medicine use is expected to reach 4.5 trillion doses in 2020 with the cost predicted being 1.4 trillion dollars. In the same year, over half of the global population will use more than one dose of medicine per person per day. Two-thirds of the medicines in the market will be used in emerging markets, India, China, Brazil, and Indonesia being the leading ones. An improvement in access to medicines globally is also expected, with differences still existing among countries [1].

United Nations Millennium Development Goals related findings reported the availability of medicines in developing countries to be low, with an average of 34.9% in the public sector of 27 developing countries. Similarly, availability in private sectors was found to be 36.8% [2]. Another study in Ghana, Kenya, and Uganda shows varying levels of availability among the countries and different levels of health institutions in the countries [3].

As to appropriateness, a World Health Organization (WHO) document on appropriate/rational prescribing outlines a six-step approach to the process. These include defining the problem with the patient, setting objectives regarding its treatment, assessing whether p-drugs/treatments could treat it (medicines physicians commonly prescribe and are familiar with), initiating the treatment, providing information, instruction, and warnings, and monitoring/stopping the treatment [4]. Another article which added two steps to the above process included consideration of cost of medicines and employing computer and other tools to minimize the occurrence of prescribing errors [5].

Looking at medicine use in Africa, a systematic review of studies done in eleven countries on medicine prescribing practice spanning over twenty years period reported poor prescribing practice. Compared against WHO's criteria, the review pointed out that high level of antibiotics and injections were prescribed. The review noted that the findings were worse compared to studies from other regions [6].

Studies on potential drug-drug interaction (PDDI) in a number of countries reported varying findings. Studies from India and Sri Lanka, which employed Medscape Interaction Checker, reported PDDIs proportions of 83.42% and 52.5% [7, 8]. Findings from Brazil and Italy, using Drug Reax of Micromedex, identified 49.7% and 45.3% pairs of medicines with PDDIs [9, 10].

In Ethiopia, a number of studies assessed availability, affordability, and appropriateness. Among these, there were studies on availability and prices of medicines in the public and private sectors [11]. Similarly, another study focused on availability of medicines at health centers in rural Ethiopia, in relation to status of protection by fee waiver system to patients from costs due to medicines [12]. Another assessment of availability and affordability of essential medicines for children in the western part of Ethiopia reported levels of 43% and 42.8% in public and private sectors, respectively [13].

As to the pattern of medicine prescribing, some studies reported a range of findings from different parts of the country [14–19]. A few studies on appropriateness of medicine prescription, in the form of assessing PDDIs, were conducted in Ethiopia. These focused on a range of patient categories including pediatrics, elderly, outpatients, and inpatients. Considerable proportions of prescription of PDDIs were reported by many of these studies [20–24].

Despite the above literature, studies assessing the pattern and appropriateness of medicines prescribed to outpatients remain scarce in providing information to stakeholders for possible actions. This study aimed to assess the pattern and appropriateness of medicines prescribed to outpatients visiting Gondar University Referral Hospital (GURH) in northwestern Ethiopia.

## 2. Materials and Methods

### 2.1. Study Setting and Period

This study was conducted at Gondar University Referral Hospital (GURH) from 2 to 20 May, 2016. It is a referral and teaching hospital with a catchment population of five million. It provides services in several departments including obstetrics and gynecology, internal medicine, surgery, and pediatrics. Inpatient as well as outpatient pharmacy services are also provided in the hospital.

### 2.2. Study Design

An institution-based cross-sectional study was conducted among adult outpatients visiting the hospital outpatient pharmacy. This was undertaken through interview with patients at the pharmacy when coming to collect prescribed medicines and reviewing their prescriptions.

### 2.3. Sampling

This manuscript presents the pattern and appropriateness of prescribed medicines; it is part of a project which focused on assessing pattern of medicines prescribed and costs incurred by patients and their families during outpatient visits to the hospital. The sample size determination was made based on estimate of costs incurred by patients. Published mean and standard deviation (SD) of cost for general outpatient visits were not found from previous studies in Ethiopia, to the best of literature search done. So, sample size calculation was done using the single population mean formula through making reasonable estimates of the incurred minimum and maximum costs. These were used to predict standard deviation (SD) which was taken to be a quarter of the range [25–27].

The cost range estimate was from 25 Ethiopian Birr (ETB) (a little more than 1 USD) to a maximum of 500 ETB (about 22 USD), SD being 118.75 ETB. The formula [*N* = ((*z*_1−∝_)^2^ × *σ*^2^)/*δ*^2^], is used to calculate the sample size, where *z*_1−∝_ was set as 1.96 at 95% confidence interval (CI), with a standard deviation (*σ*) of 5.44 USD (118.75 ETB), and margin of error (*δ*) set at 5% (0.6 USD (13.12 ETB)). This provided a sample size of 314.5 and with a 10% contingency; the total sample size was calculated to be 346.

In the selection of participants of the study, every fifth patient receiving service at the outpatient pharmacy was approached, in each of the fifteen weekdays during the data collection period by dividing total sample by the number of days. This was done to increase the representativeness of the sample of outpatients visiting the hospital.

### 2.4. Data Collection

The data collection instrument contained sections on sociodemographic variables and medicines prescribed. It was pretested on prescriptions for 30 patients before the actual data collection which were not included in the final analysis.

Data collection was conducted from 2 to 20 May, 2016, by four pharmacy students. Interviews with patients and review of prescriptions they brought to the outpatient pharmacy were conducted. The data collectors were provided with one-day training on the data collection, instrument, and communication with respondents.

### 2.5. Data Entry, Analysis, and Interpretation

The data collected were entered into and analyzed with Statistical Packages for Social Sciences version 23. Mean, median, and proportion have been employed for descriptive analysis, presented using tables and a graph. Binary logistic regression test was done to identify predictors of the occurrence of PDDIs; the results are presented using crude and adjusted odds ratio [OR]. In this analysis a 95% confidence interval (CI) with a *p* value of 0.05 was used as cut-off point to determine statistical significance of associations.

The medicines identified from the prescriptions for patients in the study were classified into level one Anatomical Therapeutic Chemical (ATC) groups [28]. Availability of medicines was calculated using the proportion of total number of medicines patients were able to get from the pharmacy to the total number of medicines prescribed. In assessing the affordability of medicines among patients, the average spending per medicine was divided by the daily wage of the lowest paid government worker [29].

The presence of potential drug-drug interactions (PDDIs) was assessed using Medscape Drug Interaction Checker, an online interaction checking platform [30]. The levels, “Serious-Use Alternative,” “Monitor Closely,” and “Minor,” were used to classify the PDDIs found among the medicines prescribed.

### 2.6. Ethical Considerations

The study was approved by the Ethical Review Committee of School of Pharmacy at the College of Medicine and Health Sciences, University of Gondar. In the process of data collection, the consent of each of the participants of the study was sought. The collected data were kept strictly confidential and used only for the purposes of the study.

## 3. Results

Of the 346 interview encounters and prescription reviews, 342 were included in the final analysis with the remaining removed due to incompleteness. Most of the participants in the study were female patients (61.1%) and were under the age of 30 (41.2%). More than a third (33.9%) of the participants reported that they were unable to read and write. In terms of occupation, a comparable proportion (35.1%) reported to having the role of housewife. The majority of the individuals in the study (69.0%) earned less than 500 ETB (22.9 USD). Two-thirds (64.9%) of them were also from outside the town of Gondar (Table 1).

In terms of the nature of patients' visit to the hospital, the majority came for treatment of a chronic illness or a follow-up visit, which accounted for two-thirds. The remaining came due to acute illness.

### 3.1. Medicines Prescribed to Patients

**Figure 1:**
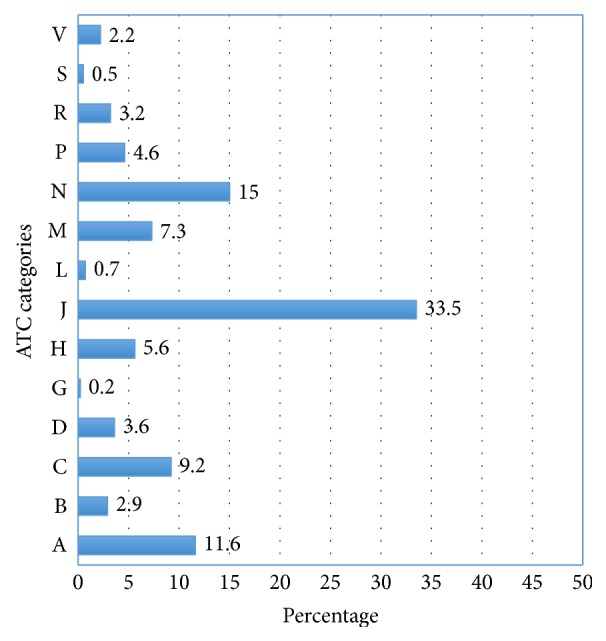
*ATC level one categories of medicines prescribed to patients*. A: alimentary tract and metabolism; B: blood and blood forming organs; C: cardiovascular system; D: dermatologicals; G: genitourinary system and sex hormones; H: systemic hormonal preparations, excluding sex hormones and insulin; J: anti-infectives for systemic use; L: antineoplastic and immunomodulating agents; M: musculoskeletal system; N: nervous system; P: antiparasitic products, insecticides, and repellents; R: respiratory system; S: sensory organs; V: various.

The outpatients visiting the hospital were prescribed 106 different medicines, with a total frequency of 588 medicines. This was calculated to be 1.72 medicines per patient on average. Looking at the type of medicines prescribed, anti-infectives for systemic use were the most frequent level one ATC groups of medicines, a third (33.5%) of the medicines prescribed. Medicines for nervous system (15.0%) and those for alimentary tract and metabolism (11.6%) followed anti-infectives in their frequency (Figure 1).

The ten most frequently prescribed medicines accounted for nearly half (44.7%) of the total, amoxicillin and omeprazole being the most common ones (Table 2).

### 3.2. Availability and Affordability of the Prescribed Medicines

Of the total number of medicines prescribed, patients were able to get their prescriptions filled for the 496 medicines (84.4%). The average amount of money per medicine, patients spent at the dispensary, was 1.45 USD. As to the affordability of the medicines dispensed to them, the average amount of money spent per medicine and the daily wage of lowest paid unskilled government employees (0.87 USD) were used to calculate it. On the basis of this, an unskilled lowest paid government employee needs to work for more than one and a half day (1.67 days) to be able to pay for a medicine prescribed to them.

### 3.3. Proportion and Predictors of PDDIs among Medicines Prescribed to Patients

Of the 342 patients in the study, 164 were prescribed two or more medicines. Among these prescription encounters, just above a third (34.5%) were found to have at least one PDDI.  Table 3 summarizes the commonest pairs of medicines involved in PDDIs. Amoxicillin and doxycycline coprescription was found to be the most common.

Looking at the severity level of the PDDIs, those requiring close monitoring due to their significance were found to account for more than half (56.31%) of the total PDDIs identified. PDDIs which were associated with a recommendation at “serious” level contributed the lowest proportion accounting for 13.59% (Table 4).

In terms of the predictors associated with the prescribing of PDDIs, male patients were more than three times likely to be prescribed medicines with PDDIs compared to women (AOR = 3.168 [1.320–7.601]). PDDIs were also associated with age, persons in the age group of 50 to 59 years being more than six times more likely (AOR = 6.457 [1.921–21.702]) to get prescriptions with PDDIs compared to patients in the age groups of 18 to 29 years. The number of medicines prescribed was also found to predict the occurrence of PDDIs, with prescribing of one more medicine being associated with a six times more likelihood of occurrence of PDDIs (AOR = 6.002 [3.068–11.739]) (Table 5).

## 4. Discussion

The study assessed the pattern of medicines prescribed and their appropriateness in terms of proportion of prescriptions with PDDIs. The average number of medicines prescribed to patients was within the acceptable standard set by WHO [31]. Many of the findings from studies in different parts of Ethiopia showed comparably higher average number of medicines per prescription, ranging from 1.82 to 2.34 [15–19, 32]. Similarly higher number of medicines per prescription were reported by studies from other countries including China, Nigeria, and United Arab Emirates [33–35]. Difference in the composition of cases among patients for which medicines were prescribed could be one of the reasons for the difference.

As to the type of medicines prescribed to outpatients, anti-infectives for systemic use were the most frequent ones. This was in line with the disease burden in the country, where many of the top ranking diseases causing disability or death are of infectious nature [36]. A study from Sri Lanka reported medicines for acid related disorders and antibacterials among the most frequently prescribed. This was similar to the current study, although the top two groups in the cited study were for noninfectious diseases [8]. Other studies in Ethiopia also reported similar findings with anti-infectives prescribed in high proportions [17–20]. Looking at individual medicines, seven of the ten most frequently prescribed medicines were anti-infectives. Among these, amoxicillin was the most frequently prescribed, similar to findings of other studies in different parts of Ethiopia [15, 17, 18].

Of the total number of medicines prescribed to them, patients were able to get about 85%. This was lower compared to the WHO recommended standards [31]. Studies in different parts of Ethiopia reported a range of findings in the different hospitals studied, ranging from as low as about 70% to 100% of the prescribed medicines actually dispensed [15–17, 19]. A study assessing health centers in rural Ethiopia reported a comparable proportion of medicines availability based on the proportion of prescriptions filled [12].

In regard to affordability of medicines, lowest paid government employee needs to work for more than a day and a half to be able to afford a medicine of average price among those prescribed. This was fairly comparable to those from other studies as it took average amount of money spent per medicine into consideration. Other studies from Ethiopia reported a range of 0.3-day to more than one-week duration of wages, for specific medicines [13]. Taking the fact that a third of the Ethiopian population lives under poverty line, despite recent improvements in the economy, the spending reported here is significant [37]. Another study from the Philippines reported similarly varying durations, from less than one day to more than four days depending on the price of medicines, for generic ones [38]. A much lower duration ranging from 48 minutes to more than one and a half hour work was found by a study from Fiji [39].

As one of the important indicators of appropriateness, presence of PDDIs among the medicines prescribed to patients was assessed. More than a third of the patients in the study were prescribed at least one pair of medicines with PDDIs. This was a little higher compared to a finding from a study in the US which assessed PDDIs in outpatient settings [40]. However, other studies from Ethiopia and other countries found higher to much higher proportions of PDDIs compared to the current study, reaching up to 78% [8, 10, 21, 22, 41]. The difference could be associated with higher number of medicines prescribed per individual in the cited studies and the fact that some were done at inpatient facilities where generally higher number of medicines are prescribed, leading to higher probability of interactions occurring.

Looking at factors related to occurrence of PDDIs, being male was statistically significantly associated with a higher likelihood. In contrast, a study among medicines prescribed for the elderly, conducted in Brazil, identified being female to be associated with occurrence of PDDIs [42]. However, a number of other studies found no statistically significant association in terms of sex of patients [21–24, 41]. Difference in specific conditions/diagnoses between male and female patients could be among the possible reasons. Alternatively, due to the smaller number of prescriptions with two or more medicines and the lower proportion of male patients, random variation might have resulted.

Another variable found to be associated with higher likelihood of prescription of PDDIs was age, specifically those aged from 50 to 59 compared to those between the ages of 18 and 29 years. Higher number of medicines and occurrence of more ailments in older ages could explain the higher likelihood of PDDIs. The finding goes in line with studies from Ethiopia, Brazil, and Serbia, which reported that prescriptions for older patients were associated with higher likelihood of PDDIs [21, 41, 42]. Some other studies reported no statistically significant association.

The mean number of medicines prescribed per prescription was associated with higher likelihood of PDDIs occurring. This was in agreement with the findings from many studies including those from Ethiopia, Brazil, and Serbia [21–24, 41, 42].

## 5. Limitation

The study considered a duration of one month which may not have covered seasonal variations in prescribing of medicines.

## 6. Conclusions

The study found out that the number of medicines per prescription to be acceptable though their availability fell short of recommended level. In terms of type of medicine, anti-infectives took the highest share among those prescribed. The medicines prescribed were also found to have a considerable cost on patients. As to appropriateness, the study also identified presence of a significant proportion of medicine pairs with PDDIs. Age and number of medicines were found to predict PDDIs.

On the basis of the finding, it is recommended that availing medicines in the dispensary should be given due attention by the hospital to help realize good patient outcome and save patients from costs incurred when buying it outside the hospital. Physicians should consider prescribing alternative medicines in terms of PDDIs, especially when dealing with patients requiring many medicines.

## Figures and Tables

**Table 1:** Sociodemographic profile of participants at GURH, 2016.

Variable	Frequency (%)
Age (years)	
18–29	141 (41.2)
30–39	84 (24.6)
40–49	46 (13.5)
50–59	45 (13.2)
60+	26 (7.6)
Gender of the patient	
Male	133 (38.9)
Female	209 (61.1)
Marital status	
Married	195 (57.0)
Unmarried	100 (29.2)
Divorced/separated	27 (7.9)
Widow/er	20 (5.8)
Educational status	
Unable to read and write	116 (33.9)
Able to read and write	33 (9.6)
Primary school (Grades 1–8)	72 (21.1)
Secondary school (9-10)	55 (16.1)
College preparatory level	14 (4.1)
Technical and vocational education and training	31 (9.1)
University education	21 (6.1)
Major occupation	
Government employee	36 (10.5)
Private company employee	18 (5.3)
Self-employed/business person	16 (4.7)
Housewife	120 (35.1)
Farmer	76 (22.2)
Student	43 (12.6)
Unemployed	21 (6.1)
Other^a^	12 (3.5)
Permanent residence	
Gondar town	120 (35.1)
Areas outside Gondar town	222 (64.9)
Monthly income	
Up to 500 ETB (22.9 USD)	236 (69.0)
More than 500 ETB (>22.9 USD)	106 (31.0)

^a^Daily laborer, driver.

**Table 2:** The ten most frequently prescribed medicines at the outpatient department of GURH, 2016.

Rank	Name of medicine	Frequency (%)
(1)	Amoxicillin	47 (8.0)
(2)	Omeprazole	46 (7.8)
(3)	Metronidazole	29 (4.9)
(4)	Doxycycline	24 (4.1)
(5)	Amoxicillin + clavulanic acid	21 (3.6)
(6)	Ciprofloxacin	21 (3.6)
(7)	Insulin	21 (3.6)
(8)	Tramadol	20 (3.4)
(9)	Diclofenac	18 (3.1)
(10)	Norfloxacin	15 (2.6)

**Table 3:** The five most frequent pair of medicines involved in PDDIs, GURH, 2016.

Pair of medicines in PDDIs	Type	Severity	Frequency (%)
Amoxicillin + doxycycline	Pharmacodynamic	Serious-Use Alternative	8 (7.77)
Aspirin + enalapril	Pharmacodynamic	Serious-Use Alternative	6 (5.82)
Furosemide + spironolactone	Pharmacokinetic	Monitor Closely	6 (5.82)
Amoxicillin + clarithromycin	Pharmacodynamic	Minor	5 (4.85)
Amoxicillin + azithromycin	Pharmacodynamic	Minor	4 (3.88)

**Table 4:** Level of severity of PDDIs identified among medicines prescribed, GURH, 2016.

Level of PDDI	Frequency (%)
Serious-Use Alternative	14 (13.59)
Monitor Closely	58 (56.31)
Minor	31 (30.10)
Total	103 (100.0)

**Table 5:** Binary logistic test for the predictors of PDDIs, GURH, 2016.

Variable	Presence of PDDI		Crude OR [95% CI]	Adjusted OR [95% CI]
	Yes (%)	No (%)		
Sex				
Male	28 (43.1)	37 (56.9)	1.827 [0.949–3.515]	3.168 [1.320–7.601]^*∗*^
Female	29 (29.3)	70 (70.7)	1	1
Age group (Years)				
18–29	13 (22.4)	45 (77.6)	1	1
30–39	14 (31.8)	30 (68.2)	1.615 [0.667–3.914]	1.223 [0.426–3.516]
40–49	8 (34.8)	15 (65.2)	1.846 [0.642–5.312]	2.181 [0.562–8.466]
50–59	13 (56.5)	10 (43.5)	4.500 [1.607–12.607]^*∗*^	6.457 [1.921–21.702]^*∗*^
60+	9 (56.3)	7 (43.8)	4.451 [1.389–14.264]^*∗*^	3.972 [0.897–17.598]
Illness/condition				
New/recently occurred	21 (39.6)	32 (60.4)	1	1
Chronic/follow-up	36 (32.4)	75 (67.6)	0.731 [0.371–1.442]	0.621 [0.263–1.466]
Number of medicines				
(mean (SD))	3.09 (1.09)	2.22 (0.48)	4.718 [2.645–8.416]^*∗*^	6.002 [3.068–11.739]^*∗*^

^*∗*^ *p*-value < 0.05.
